# CeO_2_ Enhanced Ethanol Sensing Performance in a CdS Gas Sensor

**DOI:** 10.3390/s17071577

**Published:** 2017-07-05

**Authors:** Meishan Li, Wei Ren, Rong Wu, Min Zhang

**Affiliations:** 1 The School of Physics Science and Technology, Xinjiang University, Urumqi 830046, China; meishan_li@126.com (M.L.); wurongxju@sina.com (R.W.); 2 Xinjiang Key Laboratory of Electronic Information Materials and Devices, Xinjiang Technical Institute of Physics & Chemistry, CAS, Urumqi 830011, China; renw@ms.xjb.ac.cn

**Keywords:** ethanol sensors, nanowires, CdS, CeO_2_

## Abstract

CdS nanowires (NWs) were fabricated through a facile low-temperature solvothermal method, following which CeO_2_ nanoparticles were modified on the NWs. The ethanol sensing characteristics of pure CdS and decorated ones with different CeO_2_ content were studied. It was found that the sensing performance of CdS was significantly improved after CeO_2_ decoration. In particular, the 5 at% CeO_2_/CdS composite exhibited a much higher response to 100 ppm ethanol (about 52), which was 2.6 times larger than that of pure CdS. A fast response and recovery time (less than 12 s and 3 s, respectively) were obtained as well as an excellent selectivity. These results make the CeO_2_-decorated CdS NWs good candidates for ethanol sensing applications.

## 1. Introduction

Gas sensors have been extensively applied in various fields, such as exhaled breath analysis in medical diagnosis, vehicle exhaust monitoring in environmental protection, anti-terrorism in personal and national security, etc. In recent years, sensors based on semiconductors with a one-dimensional (1D) nanostructure (in the forms of fibers, wires, sheets, rods, tubes, etc.) have been rapidly developed [1,2,3,4,5]. Compared to their micro-sized or bulk materials, 1D nanomaterials have been demonstrated with large surface specific areas, fast electron transport, and straight conduction pathways, which make them highly sensitive and efficient in detecting the target gas [6]. Various techniques have been adopted to generate 1D nanomaterials, including solvothermal routes, electrospinning, wet-chemical, hydrothermal synthesis, and the vapor phase transport method; among them, the solvothermal routes emerged as a simple and low-cost way for preparing uniform, pure, and high-quality materials [7,8].

CdS, with a direct energy gap of 2.42 eV, shows real application in various areas, such as in photoconductors, photocatalysts, laser printers, and in the paint industry [9,10]. Furthermore, CdS exhibits low resistance, strong interaction with gas molecules, high sensitivity, and fast response time [11]. The research on the gas sensing properties of CdS is limited; in particular, the metal oxide-decorated CdS with heterostructures has seldom been researched. This combination of different components surprisingly results in a synergistic effect. The response and selectivity of the sensors based on heterostructures could be improved greatly by the barrier formed at the contact interface [12]. Some research on Au-decorated CdS with Schottky junction has been reported previously, however, its response time was long and its working temperature was high, which causes the problems of high energy consumption and thermal instability [8]. As an important functional material, CeO_2_ has been used in barrier layers, capacitor devices, and as a catalyst support [2]. Also, CeO_2_ is widely adopted to enhance the sensing properties of ZnO [1], TiO_2_ [13], as well as In_2_O_3_ [5]. Therefore, sensors based on CeO_2_-decorated CdS NWs might possess more possibility in the realization of an increased sensitivity and response speed. In this paper, CeO_2_-decorated CdS NWs were fabricated by a solvothermal method. Their gas sensing properties were investigated and an excellent sensing performance towards ethanol was obtained, which makes the composite a good candidate for developing high response ethanol sensors.

## 2. Experimental Procedure

In the experiment, CdS NWs were synthesized by a facile solvothermal method. The preparation details can be found in Reference [8]. CeO_2_-decorated CdS NWs were synthesized through a similar method. Specifically, 5 mg as-prepared CdS NWs were dispersed in a mixture containing 50 mL deionized water and 30 mL ethanol. Subsequently, a measured amount of cerium nitrate Ce(NO_3_)_2_ (by mole ratio of 20:1 and 10:1) was added into the suspension under magnetic stirring. Thereafter, the solution was transferred into a Teflon-lined stainless-steel autoclave with a capacity of 100 mL. The autoclave was kept at 180 °C for 24 h. Finally, the product was collected after the centrifugation and drying processes. The sample prepared above was mixed with deionized water in a weight ratio of 100:4 to form a paste. The paste was then coated on an alumina ceramic tube, on which a pair of Au electrodes formed the sensing film. Pt wires wedged on the pedestal were used as electrical contacts and a Ni–Cr wire was inserted through the tube using as a heater to control the operating temperature. The crystal structure of the samples was examined by X-ray diffraction (XRD, Bruker, D8 Advance, Karlsruhe, Germany). A scanning electron microscope (SEM) (LEO1430VP, Zeiss, Jena, Germany) was used to investigate the morphology. Gas sensing properties were measured by CGS-8 intelligent gas sensing analysis system (Beijing Elite Tech Co., Ltd., Beijing, China). The response value was defined as R_a_/R_g_, where R_a_ and R_g_ were the resistance of sensors in air and their presence in the target gases, respectively.

## 3. Results and Discussion

**Figure 1:**
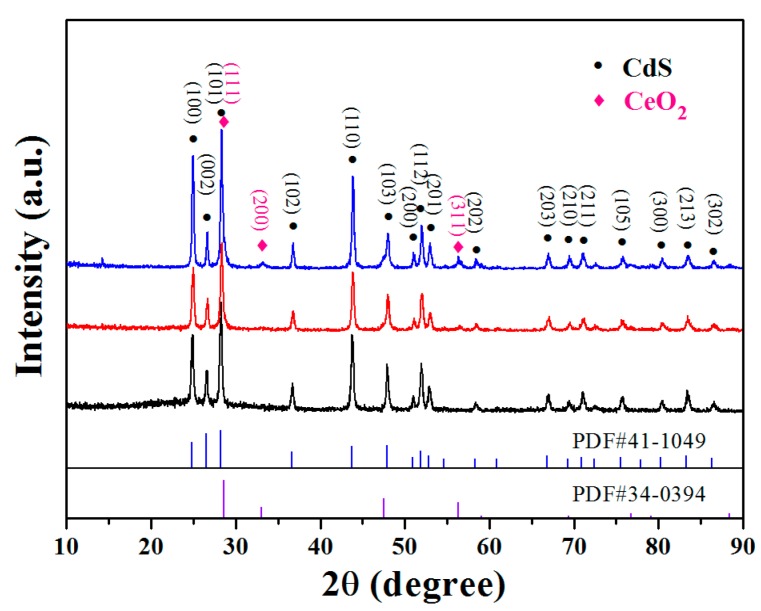
The XRD patterns of pure CdS NWs and CeO_2_/CdS composites with different CeO_2_ contents.

The XRD patterns of as-prepared pure CdS and CeO_2_-decorated ones are shown in Figure 1. The diffraction peaks (111), (200), and (311) in the spectrum can be indexed to be CeO_2_ (JCPDS 34-0394); while (100), (101), (110) are the main reflections of CdS (JCPDS 41-1049). The XRD analysis of CeO_2_/CdS NWs reveals the presence of both cubic-structured CeO_2_ and hexagonal-typed CdS in the composite. No diffraction peaks from other impurities were found, indicating the high purity of the samples.

**Figure 2:**
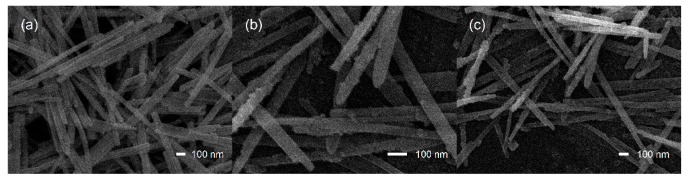
SEM images of CdS NWs (**a**) and the composites with high (**b**) and low (**c**) CeO_2_ content.

Figure 2a displays the general morphology of pure CdS NWs studied with SEM, which indicates the formation of a 1D wire-like nanostructure with diameter of about 30 nm. The structure feature of the CeO_2_-decorated CdS is shown in Figure 2b,c. It is evident that the composite still keeps the original morphology as CdS NWs. Under the same magnification, more adhesions on the CdS surface were discovered after CeO_2_ decoration.

**Figure 3:**
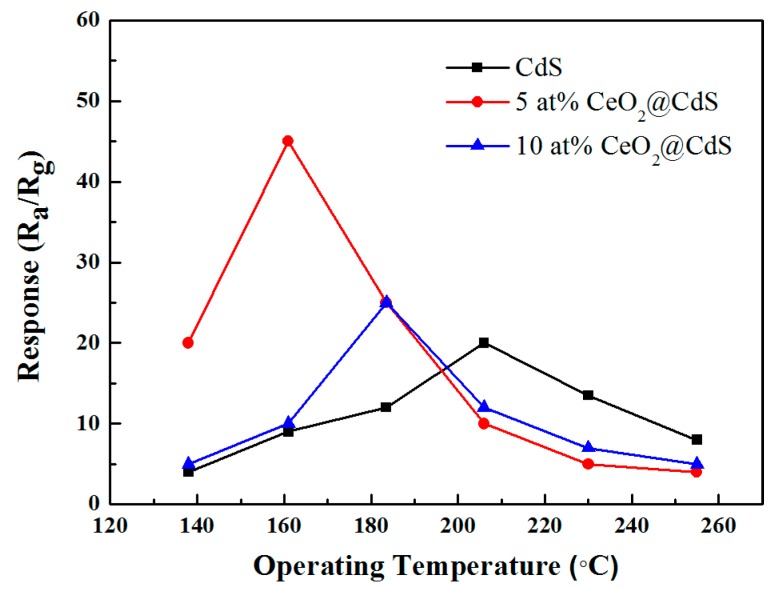
Response of CdS NWs and CeO_2_/CdS composites to 100 ppm ethanol vapor measured at different working temperatures from 138 to 255 °C.

In order to determine the optimum working temperature, the response of CdS and CeO_2_/CdS NWs to 100 ppm ethanol was measured at different temperatures (138–255 °C). As shown in Figure 3, the response of CdS NWs to ethanol vapor increased with the working temperature and achieved the maximum value at about 206 °C, followed by a decrease with the temperature. As for CeO_2_-decorated ones, 5 at% CeO_2_/CdS NWs showed an optimum working temperature of 161 °C, while that of the 10 at% composite was about 183 °C. Ma and co-workers reported Au-decorated CdS prepared by the same method as ours, which worked best around 220 °C with a peak response of 110 [8]. Deng and Liu et al. recently investigated the enhanced ethanol sensing properties of ZnO-based composites, and both of the optimum working temperatures were higher than 220 °C [14,15]. In contrast to this past research, the presented CeO_2_/CdS NWs exhibit a much lower working temperature. This improves the security and reduces the energy consumption for the sample in device application. However, the response of CeO_2_/CdS NWs still has room to be strengthened. In addition, at the optimum working temperature, the 5 at% composite shows notably higher sensitivity than the pure sample, indicating that the decoration of CeO_2_ on CdS NWs is beneficial for the enhancement of ethanol detection.

**Figure 4:**
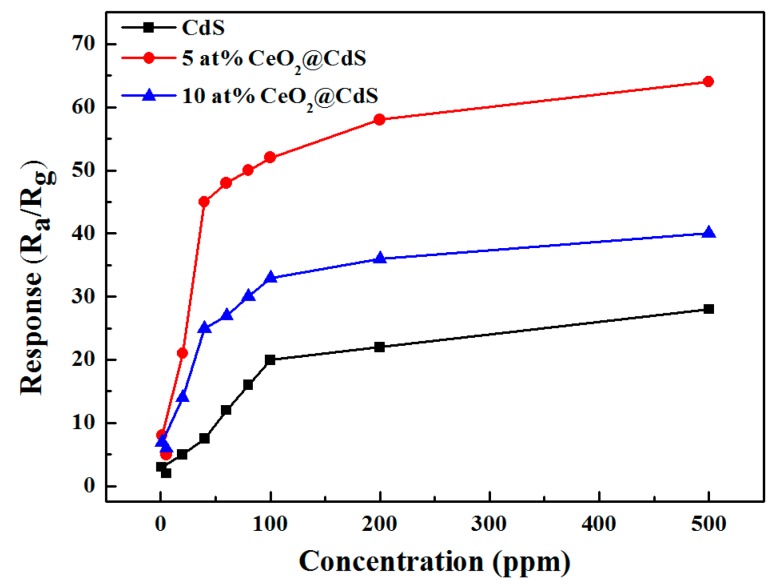
The response of the sensors versus different ethanol concentrations from 1 to 500 ppm at the optimum working temperature.

The response of the sensors versus different ethanol concentrations at the optimum working temperature is shown in Figure 4. The response rises upon increasing the ethanol concentration from 1 to 200 ppm. The response increase slows down and gradually saturates above 200 ppm. Moreover, the 5 at% CeO_2_/CdS NWs display a much higher response with the maximum value of about 52 to 100 ppm ethanol vapor (2.6 times larger than that of pure CdS).

**Figure 5:**
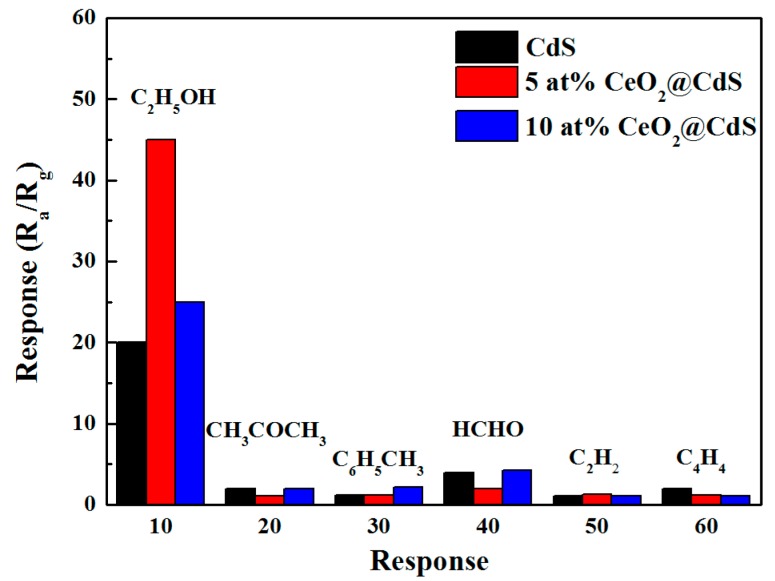
Selectivity of the sensors to 100 ppm ethanol (C_2_H_5_OH), acetone (CH_3_COCH_3_), toluene (C_6_H_5_CH_3_), formaldehyde (HCHO), ethyne (C_2_H_2_), and tetrahedrane (C_4_H_4_).

The selectivity of a gas sensor is one of the important parameters to evaluate in the sensing performance of semiconductors. The response of pure CdS NWs and CeO_2_-decorated NWs to different gases is shown in Figure 5. The detected gases are ethanol (C_2_H_5_OH), acetone (CH_3_COCH_3_), toluene (C_6_H_5_CH_3_), formaldehyde (HCHO), ethyne (C_2_H_2_), and tetrahedrane (C_4_H_4_) with a concentration of 100 ppm, respectively. As shown in this figure, the composite sensors exhibit a much larger response to ethanol than to other interfering gases. On the one hand, CdS detects ethanol efficiently by catalyzing the decomposition of ethanol molecules. On the other hand, the decoration of CeO_2_ would effectively dehydrogenate ethanol molecules and then significantly improve the selectivity towards ethanol.

A similar material system as we used, composed of In_2_O_3_-decorated ZnS NWs, also acquired higher sensitivity and lower operating temperature than the pristine sensor [16]. In the research work by Choi, Cr_2_O_3_-functionalized WO_3_ with p-n junction was facilely synthesized by coating Cr_2_O_3_ on the nanorods substrate. However, the sensors in these works not only detect ethanol well, but also show a response to acetone as well as other gases [17]. A comparison between CeO_2_/CdS NWs and these composites indicates the excellent selectivity of the 5 at% sample for detecting low concentrations of ethanol.

**Figure 6:**
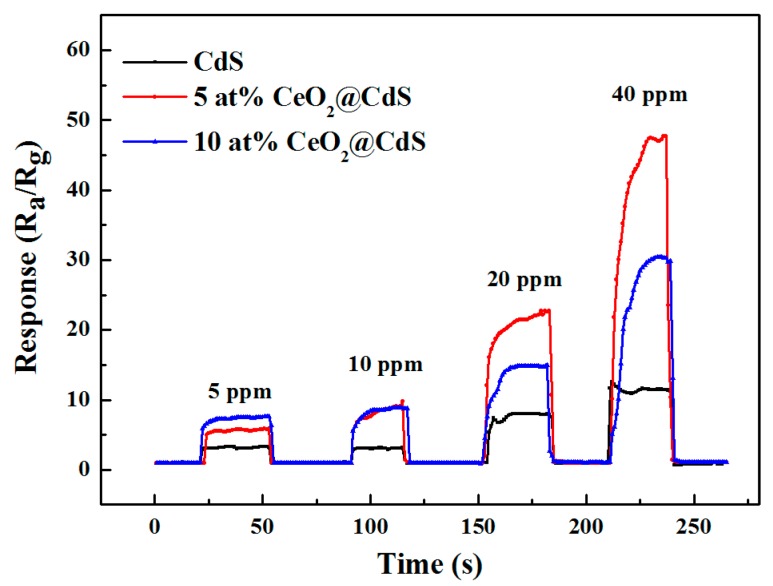
Response and recovery behaviors of CdS NWs and CeO_2_/CdS composites to different concentrations of ethanol (5 ppm, 10 ppm, 20 ppm, 40 ppm).

The response time is the time taken by a sensor to achieve 90% of the total resistance change in the case of the adsorption process, or the recovery time in the case of the desorption process. The response and recovery characteristics of the sensors to different concentrations of ethanol are shown in Figure 6. It is noted that when the detected gas is injected into the testing chamber, the response magnitude ascends with the increase of ethanol concentration, followed by a drop with the absence of the target gas. All the sensors display a fast response for ethanol detection, and both the response and recovery time of CdS NWs are less than 3 s. Those of 5 at% CeO_2_/CdS NWs are less than 12 s and 3 s, respectively, which is much shorter than that of other CdS and decorated metal oxides-based ethanol sensors [18,19].

When the ethanol concentration is as high as 40 ppm, the composite with fewer CeO_2_ adhesions shows a higher response. On this occasion, too many CeO_2_ particles would occupy the reactivity sites on the CdS surface, which in turn lowers the response. While at low ethanol levels, the active site is adequate in the chemical reaction, and the impact of CeO_2_ content on the response is not obvious. Compared with the one-dimensional structured CdS NWs, the longer response time of composites is possibly ascribed to the numerous grain boundaries generated after CeO_2_ decoration, which slows down the electron transport.

The most widely accepted sensing mechanism is based on the resistance change on the surface of a sensitive film by the adsorption and desorption of gas molecules. When the semiconductors are surrounded by air, oxygen molecules are adsorbed onto the surface by chemisorption. Then, oxygen species (O_2_^−^, O^−^) are formed by extracting electrons from the conduction band of a semiconductor, which leads to the formation of an electron-depleted layer on the surface and a decreased conductance in the semiconductor. When the semiconductors are exposed to the reducing gas ethanol, the gas molecules react with the chemisorbed oxygen and release the trapped electrons back to the conduction band of the semiconductor, which narrows the depletion layer width and results in the resistance reduction in the sensors [6]. The overall reaction may take place as follows [20]:

O_2(gas)_ → O_2(ads)_ (1)

O_2(ads)_ + e^−^ → O_2_^−^_(ads)_ (T<100 °C) (2)

O_2_^−^_(ads)_ + e^−^ → 2O^−^_(ads)_ (100 °C<T<300 °C) (3)

O^−^_(ads)_ + e^−^ → O^2−^_(ads)_ (T>300 °C) (4)

C_2_H_5_OH (gas) + O^−^ (ads) → CH_3_CHO + H_2_O + e^−^ (5)

The 1D nanostructure with a large surface area-to-volume ratio and straight conduction pathways contributes a lot to the high sensitivity and quick response of the current NWs, by promoting the efficient ethanol absorption on NWs surface. Another reason for the sensing improvement is that CeO_2_ acts as catalyst for the oxidative dehydrogenation of alcohols. The decorated CeO_2_ nanoparticles on the surface involve more ethanol molecules in the reaction by catalyzing them into acetaldehyde [21]. The CeO_2_/CdS NWs combine the advantages of both components and, consequently, a synergistic effect between them makes the composite more sensitive than the simplex CdS [22]. Additionally, CeO_2_ decoration induces the formation of heterojunction at the CeO_2_/CdS interface. The increased barrier height hinders the electron transmission and raises the atmospheric resistance in the composite. When exposed to ethanol, the barrier height of the composite is further reduced by the reaction between the gas molecules and the adsorbed oxygen. Then, the electrons would penetrate through the barrier easily and bring about a sharp drop in resistance. As a result, surface chemical processes are converted into electrical signals and a high response is eventually obtained [1,23].

A thermally activated surface oxidation process may be responsible for the temperature dependence of CeO_2_-decorated CdS NWs. As the temperature rises from 138 °C to 161 °C, the formation of chemisorbed oxygen species is accelerated and the reaction between these oxygen irons and ethanol is promoted. As a result, the response goes up with the operating temperature. However, as the temperature ascends further, the gas molecules quickly diffuse and desorb from the surface before involved in the reaction [24,25]. Accordingly, the response declines above the optimal temperature. Moreover, the decoration of CeO_2_ reduces the activation energy in the reaction, and then the optimum working temperature is lowered in comparison to that of pure CdS [11]. We also discovered the saturability characteristic of the samples towards a high concentration ethanol, as shown in Figure 3. In this case, the gas molecules are excessive, and limited oxygen species are involved in the reaction completely. On the other hand, the oxygen species are adequate at a low ethanol concentration, hence the response increases sharply. 

## 4. Conclusions

In conclusion, the synthesis and characterization of CeO_2_/CdS NWs were confirmed by XRD and SEM. Thereafter, a sensor based on the composite was fabricated and its ethanol sensing properties were explored for the first time. It was found that CeO_2_ decoration enhances the response and decreases the working temperature of CdS NWs. In comparison with pure CdS, the 5 at% CeO_2_/CdS NWs show an improved sensitivity to 100 ppm ethanol vapor, favorable selectivity, and fast response rate at the optimal operating temperature of 161 °C. These results suggest that CeO_2_/CdS NWs with heterostructures are of importance to the fabrication of optimized gas sensors.
